# 

**Published:** 1917-07-28

**Authors:** 


### Bombs on a "Magazine."

THE first edition of that entertaining publication "Sea Pie," which for the small outlay of 1s. 6d. enables members of the public to benefit British naval prisoners in the hands of the enemy, was in danger of being destroyed when bombs were dropped, during the last air raid, on the printing offices. Although much damage was done by fire and water, the greater part of the edition was saved for the amusement of the public and the good of the naval prisoners.

### Passing Events and Topics.

### Prince of Wales' Hospital for Limbless Sailors and Soldiers, Cardiff.

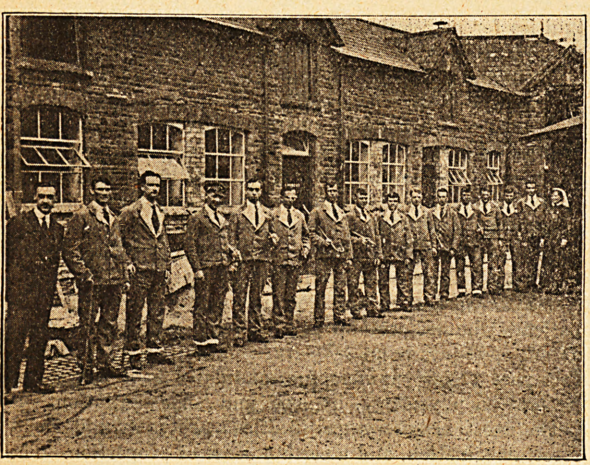

*Photo*] [*Welsh Pictorial.*

THE SECOND BATCH OF PATIENTS RECEIVED AT THE PRINCE OF WALES' HOSPITAL, CARDIFF.

THE above illustration represents the second batch of patients of the Prince of Wales' Hospital, Cardiff, to be fitted and discharged with artificial limbs and appliances. The photograph is taken in the laundry yard, and includes Mr. W. A. Blatchford, who is in charge of the limb workshops, and the Commandant, Miss C. Deacon. Since the hospital was opened on May 7 over twenty patients have been fitted and discharged with limbs, and the men themselves express great satisfaction with the care taken to deal with their individual cases.

### Hampstead Hospital Ladies' Association.

A REMARKABLY successful Garden Fête and Theatrical Entertainment, organised by the ladies of the Hampstead General and North-West London Hospital Association in aid of the funds of the hospital, was held recently at "The Hill," Hampstead Heath, the residence of Lord Leverhulme. It was opened by the Countess Torby, and included music, a dramatic performance by distinguished artists, organised by Mr. Gerald Du Maurier; a children's pastoral movement to music by Grieg, written and produced by Miss Silvia Hobday; and juvenile sports and races, prizes for which were provided by Lord Leverhulme and presented by Mrs. Gerald Du Maurier.

### U.S. Red Cross Fund.

THE latest reported total receipts of the campaign for the U.S. Red Cross was £20,062,600.

### War on the Casual.

THE municipal authorities up and down the country are at length taking steps to curtail the liberties and privileges of that feckless individual—the tramp. In normal times, doubtless, a fair case could be made in favour of providing abundant accommodation for the shifting and vagrant element—the true submerged tenth—which our uneconomic methods of labour distribution rendered an inevitable component of the population. But now that the labour market is clamouring for all the hands that can be obtained, the task of rendering the tramp's life a happy one is grossly unpatriotic. The fact that only the smallest proportion of these gentry fail to perform the allotted task shows that we have in them a fund of energy that has been practically untapped for national purposes. The Staffordshire Guardians, with that common-sense which we have learned to expect from them, are uniting with Warwickshire and Worcestershire in a joint movement to close down a great number of their casual wards, leaving only one within each radius of sixteen miles. "It is certain, however," adds their recently published report, "that the habitual vagrant will not reconcile himself to a useful occupation until he is thoroughly convinced that all avenues for a lazy life are closed to him."

RATES OF SUBSCRIPTION: Twelve months, 6/6; Six months, 4/-; three months, 2/-, post free to any address in the United Kingdom. Abroad, Twelve months, 8/8; Six months, 5/-; Three months, 2/6, post free. **THE HOSPITAL "Index" to Volume LXI., October 1916 to March 1917, is now ready, price 2½d. per copy, post free.** Address The Manager, THE HOSPITAL, "The Hospital" Building, 28/29 Southampton Street, Strand, London, W.C. 2.

